# Studying wedge factors and beam profiles for physical and enhanced dynamic wedges

**DOI:** 10.4103/0971-6203.57116

**Published:** 2010

**Authors:** Misbah Ahmad, Amjad Hussain, Wazir Muhammad, Syed Qaisar Abbas Rizvi, Matiullah

**Affiliations:** Institute of Nuclear Medicine Oncology and Radiotherapy (INOR), Abbottabad; Institute of Radiotherapy and Nuclear Medicine (IRNUM), Peshawar, Pakistan; 1 TBCC, Department of Medical Physics, 1331-29 street, NW Calgary, AB, Canada; 2 Department of Physics, Kyungpook National University, Daegu 702-701, Republic of Korea; 3 Institute of Nuclear Medicine Oncology and Radiotherapy (INOR), Abbottabad, Pakistan; 4 Head NTSG, Pakistan Institute of Nuclear Science and Technology (PINSTECH), Nilore, Islamabad, Pakistan

**Keywords:** Beam profile, enhanced dynamic wedge, physical wedge, wedge factor

## Abstract

This study was designed to investigate variation in Varian's Physical and Enhanced Dynamic Wedge Factors (WF) as a function of depth and field size. The profiles for physical wedges (PWs) and enhanced dynamic wedges (EDWs) were also measured using LDA-99 array and compared for confirmation of EDW angles at different depths and field sizes. WF measurements were performed in water phantom using cylindrical 0.66 cc ionization chamber. WF was measured by taking the ratio of wedge and open field ionization data. A normalized wedge factor (NWF) was introduced to circumvent large differences between wedge factors for different wedge angles. A strong linear dependence of PW Factor (PWF) with depth was observed. Maximum variation of 8.9% and 4.1% was observed for 60° PW with depth at 6 and 15 MV beams respectively. The variation in EDW Factor (EDWF) with depth was almost negligible and less than two per cent. The highest variation in PWF as a function of field size was 4.1% and 3.4% for thicker wedge (60°) at 6 and 15 MV beams respectively and decreases with decreasing wedge angle. EDWF shows strong field size dependence and significant variation was observed for all wedges at both photon energies. Differences in profiles between PW and EDW were observed on toe and heel sides. These differences were dominant for larger fields, shallow depths, thicker wedges and low energy beam. The study indicated that ignoring depth and field size dependence of WF may result in under/over dose to the patient especially doing manual point dose calculation.

## Introduction

Wedges are commonly used as beam-modifying devices in radiation therapy to optimize the target volume dose distribution. In this context, variety of wedge filters is available with modern linear accelerators. Varian's CLINAC 2100C provides two types of wedge filters; these are physical wedges (i.e. 15°, 30°, 45° and 60°) and enhanced dynamic wedges (i.e. 10°, 15°, 20°, 25°, 30°, 45°, and 60°). The physical wedges (PW) can be inserted in the treatment head of linear accelerator in four different orientations (in, out, left and right). In case of Enhanced Dynamic Wedges (EDW), the required dose distribution can be achieved by one of the collimator jaws motion in two different directions (in and out). The radiation beam intensity decreases when a wedge filter is placed in the path of it. This decrease is taken into account in calculating the treatment dose in terms of Wedge Factor (WF). It is the ratio of doses at a reference depth with and without wedge for identical field size under similar experimental conditions.[1]

Most of the time a single wedge factor is used for the treatment time/monitor units (MUs) calculation of the patients, which is usually measured for a reference field size (i.e. 10 × 10 cm^2^) and reference depth (i.e. *d*_max_ or d_10_). If the depth and field size dependency of wedge factor are not taken into account in these calculations, it may result in significant tumor-dose discrepancies for the patients.[2] It is therefore important to specify any changes to wedge factor resulting from depth and field size variations.

Physical wedges have been the primary means of producing the wedged fields. The required wedged dose profile can also be achieved by the computer control motion of one of the collimator jaws. Such type of wedge is called dynamic wedge (DW).[3] The concept of DW was introduced first time by Varian in the early 1990s in linear accelerators[4] and now almost all manufacturers of linear accelerators provide the facility of DW. The DW can provide wedge angles of 15°, 30°, 45°, 60° only for symmetric field size up to 20 cm width. The capabilities of DW are significantly improved by introducing the concept of Varian's EDW. Now, the EDW provides wedge angles of 10°, 15°, 20°, 25°, 30°, 45°, and 60° for both symmetric and asymmetric field sizes up to 30 cm width. Although both the PWs and EDWs generate the same dose distribution they are expected to have some different dosimetric characteristics due to the use of different mechanism and their relative position in the treatment head of linear accelerator. The dosimetric characteristics of Siemen's virtual and physical wedges have been studied.[5,6] A number of studies have been conducted on PWs[1,2,7] and EDWs.[8–11] However, so far studies concerning comparison of Varian's PW and EDW has not been reported. The purpose of this study is to compare the WF and beam profiles for Varian's PWs and EDWs.

## Material and Methods

Comparison of WF and beam profiles of PW and EDW and its dependence on depth and field size was studied for 6 and 15 MV wedged photon beams produced by a linear accelerator, Clinac 2100 C (Varian Medical Systems) installed at Institute of Nuclear Medicine, Oncology and Radiotherapy (INOR) Abbottabad Pakistan. In the present study 15°, 30°, 45° and 60° physical and enhanced dynamic wedges were used. The 15° and 30° PW were made of Fe (cold-rolled steel) with nominal density of 7.8 g/cm^3^ whilst 45° and 60° were made of Pb (lead-calcium-tin alloy) with nominal density of 11.3 g/cm^3^. The measurement was performed for all PW and EDW of same degree in 3D Blue water Phantom (480 mm × 480 mm × 410 mm). The positional accuracy of the dosimetry system was plus/minus 0.5 mm per axis and reproducibility was plus/minus 0.1 mm. Temperature and pressure correction was applied to the measurement.

### Wedge Factor Measurement

The WF at depth *d,* in water phantom, for a field size (FS)*,* along the central axis of the beam was calculated with the help of equation 1.

(1) $$WF(FS,d)=\frac{D_{w}(FS,d)}{D_{O}(FS,d)}$$

where D_W_ (FS, d) is the dose at a specified point “d” along the central axis in a specified field size “FS” with the wedge in place and D_o_ (FS, d) is the dose at the same point in an open field of equal dimensions for the same time or number of MU.[2]

The measurements were carried out using FC65-G farmer type cylindrical water-proof ionization chamber in combination with CU500E control unit at various depths (from d_max_ to 25 cm) at a fixed field size (i.e. 10 × 10 cm^2^) in order to study depth dependency of WF. While for field size dependency of WF the measurements were made for different field sizes, varying from 4 × 4 cm^2^ up to 25 × 25 cm^2^, at a fixed depth of 10 cm. For the dependency of WF both with depth and filed size ionization was measured using 100 MUs at a dose rate of 320 MU/min and fixed SSD.

The normalized wedge factor (NWF) was introduced to circumvent large differences between wedge factors for different wedge angles defined as the ratio of wedge factor at a given depth and field size to the wedge factor at the normalization point, as given in equation 2 and 3 for depth dependence of WF and Field size dependence of WF respectively.

(2) $$NWF(10\times 10,d)=\frac{WF(10\times 10,d)}{WF(10\times 10,d_{nor})}$$

Where WF (10×10, d) is wedge factor for 10 × 10 cm^2^ field size at any depth “d”, and WF (10 × 10, d_nor_) is the wedge factor for 10 × 10 cm^2^ field size at the normalization point. The normalization point is d_max_ for each beam under study.

(3) $$NWF(FS,10cm)=\frac{WF(FS,10cm)}{WF(FS_{nor},10cm)}$$

Where WF (FS, 10cm) is wedge factor for any field size at 10 cm depth and WF (FS_nor,_ 10cm) is the wedge factor for 10 cm depth at the normalization field size. For depth dependence, the normalization point was the point of maximum dose (d_max_) whilst for field size dependence; the normalization field size was 10 × 10 cm^2^. To minimize the errors in the experimental values, data was taken for two wedge directions and the average of those measurements was taken as WF.

### Beam Profiles

The Scanditronix-Wellhofer LDA-99 linear detector array, having 99 high resolution p-type semiconductor detectors was used for measuring the wedge beam profiles. A multi channel dosemeter emXX connects the chamber array to an interface board in a personal computer. The electrometer has 99 channels for the chambers on the detector array. An additional 100^th^ channel is used for a reference chamber, which should be connected separately and is not attached to the detector array. With a single measurement, the LDA-99 linear array captures 99 data points at five mm intervals. Computer controlled longitudinal movement by user-defined distance increases the resolution up to one mm or better if needed. Therefore, one- or two-dimensional dose distributions can be measured very quickly. Data presented in this study were measured using a spacing of one mm for a number of field sizes and depths.

## Results and Discussion

### Depth Dependence of WF

**Figure 1:**
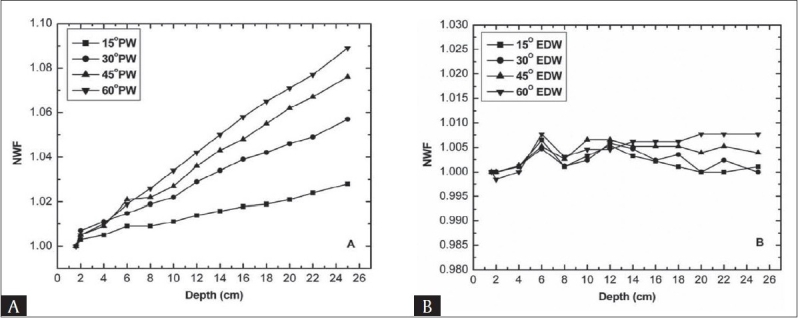
Normalized wedge factor as a function of depth for 6 MV beam at 10 × 10 cm^2^ field size (A) Physical wedge (B) Enhanced dynamic wedge

Figure 1 shows the experimentally observed normalized wedge factor (NWF) as a function of depth for four physical and enhanced dynamic wedges, using fixed field size (i.e. 10 × 10 cm^2^) for 6 MV photon beam. A gradual increase in NWF with increasing depth and wedge angle for all four physical wedges was observed [Figure 1A]. The variation in WF at different measurement depths and percentage difference in WF from d_max_ at different depths is presented in Table 1. It can be seen that the percentage difference from *d_max_* is about equal to or less than 1.1%, 2.2%, 2.7% and 3.4% up to 10 cm depth for 15°, 30°, 45°, and 60° PW and reaches to 2.8%, 5.7%, 7.6% and 8.9% for 15°, 30°, 45° and 60° wedge at 25 cm depth respectively.

**Table 1:** Percentage variation of PWF at different depths from PWF at d_max_

*Depth*	*% Difference of WF at different depths from WF at d_max_*							
	
*(cm)*	*6MV*				*15 MV*			
		
	*15° WF^1^=0.923*	*30° WF=0.849*	*45° WF=0.763*	*60° WF=0.65*	*15° WF=0.941*	*30° WF=0.881*	*45° WF=0.810*	*60° WF=0.771*
4	0.5	1.1	0.9	1	0.2	0.5	0.7	0.7
6	0.9	1.5	2.1	1.9	0.6	0.9	1.3	1.4
8	0.9	1.9	2.2	2.6	0.9	1.1	1.4	1.7
10	1.1	2.2	2.7	3.4	1	1.2	1.6	1.9
12	1.4	2.9	3.6	4.2	1.2	1.5	1.8	2.1
14	1.6	3.4	4.3	5	1.3	1.7	2	2.3
16	1.8	3.9	4.8	5.8	1.4	1.9	2.1	2.5
18	1.9	4.2	5.5	6.5	1.5	2	2.4	2.7
20	2.1	4.6	6.2	7.1	1.7	2.3	2.8	3.3
22	2.4	4.9	6.7	7.7	1.8	2.3	3.2	3.6
25	2.8	5.7	7.6	8.9	1.9	2.5	3.5	4.1

WF is the wedge factor measured at 10×10 cm^2^ field size at the depth of maximum dose.

**Figure 2:**
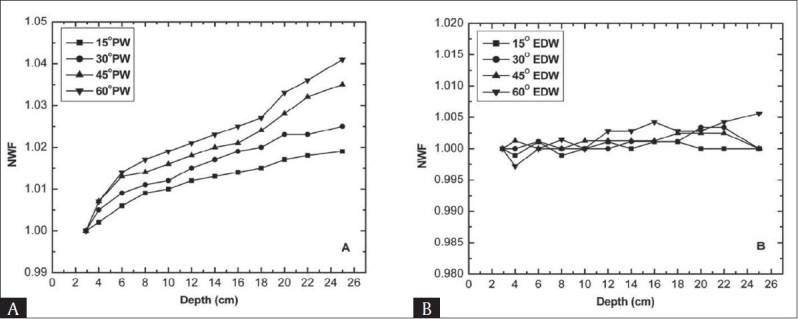
Normalized wedge factor as a function of depth for 15 MV beam at 10 × 10 cm^2^ field size (A) Physical wedge (B) Enhanced dynamic wedge

For 15 MV beam the plot of NWF versus depth for both types of wedges is shown in Figure 2. The normalization point was *d_max_* (2.9 cm). Unlike 6 MV photon beam for 15 MV photon beam small variation of NWF with depth was observed for PW. Starting from *d_max_* up to 10 cm depth the variation in NWF is less than two per cent for all four wedges. The deviation at 25 cm depth increases to 1.9%, 2.5%, 3.5% and 4.1% for 15°, 30°, 45° and 60° wedges respectively [Table 1]. The results obtained for EDW factor are quite different from PW. Unlike physical wedges, EDW factor does not changes significantly with depth and less than two per cent variation in the WF with depth were observed for all wedges at both the studied energies [Figure 1B and 2B] due to lack of beam hardening effect. The absolute values of percentage difference of EDW factor at different depths from EDW factor at dmax is presented in Table 2, showing insignificant variation. This offers a distinct advantage of EDWs over PWS. In the absence of beam hardening the small change in the EDW factor with depth is probably due to the energy fluence imbalance across the wedge direction and dose gradient scatter.[5]

**Table 2:** Percentage variation of EDWF at different depths from EDWF at d_max_.

*Depth*	*% Difference of WF at different depths from WF at d_max_*							
	
*(cm)*	*6MV*				*15 MV*			
		
	*15° WF^1^=0.923*	*30° WF=0.849*	*45° WF=0.763*	*60° WF=0.65*	*15° WF=0.941*	*30° WF=0.881*	*45° WF=0.810*	*60° WF=0.771*
4	0.108	0.118	0.131	0.000	-0.106	0.000	0.123	-0.281
6	0.650	0.471	0.524	0.769	0.106	0.114	0.000	0.000
8	0.108	0.118	0.262	0.308	-0.106	0.000	0.000	0.141
10	0.325	0.236	0.655	0.462	0.000	0.000	0.123	0.000
12	0.542	0.589	0.655	0.462	0.106	0.000	0.123	0.281
14	0.325	0.471	0.524	0.615	0.000	0.114	0.123	0.281
16	0.217	0.236	0.524	0.615	0.106	0.114	0.123	0.422
18	0.108	0.353	0.524	0.615	0.106	0.114	0.247	0.281
20	0.000	0.000	0.393	0.769	0.000	0.341	0.247	0.281
22	0.000	0.236	0.524	0.769	0.000	0.341	0.247	0.422
25	0.108	0.000	0.393	0.769	0.000	0.000	0.000	0.563

WF is the wedge factor measured at 10×10 cm^2^ field size at the depth of maximum dose.

The increase in PW factor with depth mainly arises from the beam hardening i.e. the low energy photons are attenuated much more than the high-energy photons. The beam hardening effect is dependent on the wedge material and photon energy. The 45° and 60° wedges showed larger variation in the WF as the high density material attenuates the photon flux much more than the low density material that causes an increased variation in the WF. As may be seen in Figures 1A and 2A, increase in NWF in case of low energy beam (6 MV) is more prominent as compared to high-energy beam (15 MV). It is due to the fact that 6 MV, beam hardening is higher than that for 15 MV photon beam. Therefore, at larger depths the dose values for wedge and open beam lead to a ratio that increases with depth.[2] R. C Tailar and McCullough *et al* have suggested that for energies equal to or less than 10 MV the depth dependence of WF is significant only for 45° and 60° wedges[2,12] however, our data showed that the WF variation with depth is also significant for 30° wedge. This may possibly be due to two reasons first: wedge material used in their study was different than ours and the second reason is that these values are machine specific, as given by McCullough *et al*.

### Field Size Dependence of WF

**Figure 3:**
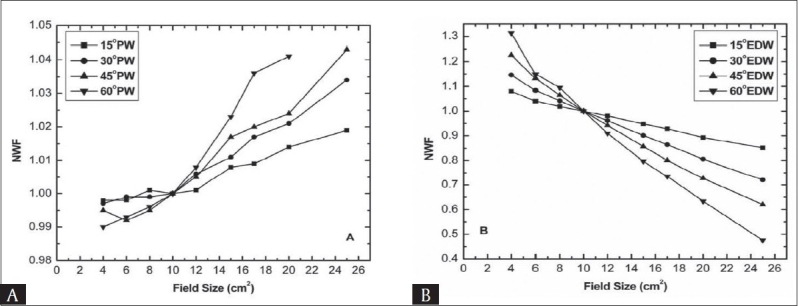
Normalized wedge factor as a function of field size for 6 MV beam at 10 cm depth (A) Physical wedge (B) Enhanced dynamic wedge

**Figure 4:**
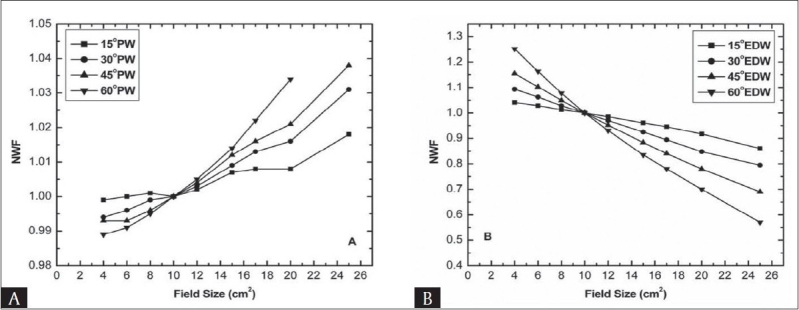
Normalized wedge factor as a function of field size for 15 MV beam at 10 cm depth (A) Physical wedge (B) Enhanced dynamic wedge

Figures 3 and 4 show experimentally observed normalized wedge factor as a function of field size at fixed depth of 10 cm for 6 MV and 15 MV photon beam respectively. No obvious increasing trend was observed with increasing field size, compared to depth dependency of NWF for physical wedges [Figures 3A and 4A]. The percentage variation of WF from WF at reference field size (i.e. 10 × 10 cm^2^) for 15° physical wedge is less significant (i.e. less than two per cent) for both energies, while for 30°, 45°, and 60° wedges up to the maximum field size, the variation is 2.1%, 2.4%, and 4.1% for 6 MV and 1.6%, 2.1%, and 3.4% for 15 MV.

Also it can be seen that the variation in NWF is less definite for field sizes less than 10 × 10 cm^2^. The field size dependence of wedge factors may be attributed due to the introduction of non-uniform scattering of photons in the presence of the wedges. In other words the dependence of wedge factor on field size is mainly due to change in the phantom and collimator scattering due to the presence of physical wedges. The scattered photon fluence increases with the irradiated wedge volume that increases with the field size. The nonlinearity with field size may be due to the fact that the scattering is not from a point, rather from within the wedge volume.

Figure 3B and 4B display the field size dependence of NWF for 15°, 30°, 45° and 60° enhanced dynamic wedges. Unlike conventional physical wedges, the EDW revealed strong dependency between field size and NWF. There seems a smooth and continuous decrease in the NWF with increasing field size. The decrease in NWF with field size is more apparent for thicker wedge, while for thinner wedges the decrease is comparatively less apparent but still significant (i.e. greater than two per cent) for all wedges at the studied energies. There is a great difference in the absolute values of PWF and EDWF. Using PW the variation in NWF with field size is less than five per cent for all wedges and both energies. whilst with EDW, the maximum variation relative to reference field size of 10 × 10 cm^2^ was from 14.75% to 51.65% from thinner to thicker wedge at 6 MV and 14.42% to 41.65% for 15 MV photon beams respectively.

The decrease in EDWF with field size can be explained from the fact that in general the exact progression of dose rate and jaw speed, as well as the total dose delivered as an open field depends on wedge angle, field size and monitor units. Since EDW uses variable dose rate and the jaw speed, which contribute high doses to the “toe” side of the wedge field with increase in field size and consequently the central axis accumulated dose decreases which causes a decrease in the central axis wedge factor. Gibbons and Vassy have also developed a model for enhanced dynamic wedge; according to that model the dose contribution to the calculation point (field chamber) is due to additional MU in the “toe” side of the wedge. It is pointed out that the number of additional MU on the toe side increases significantly with the field size as compared to the central axis, which causes a decrease in the wedge factor for enhanced dynamic wedge.[9]

### Beam Profiles

**Figure 5:**
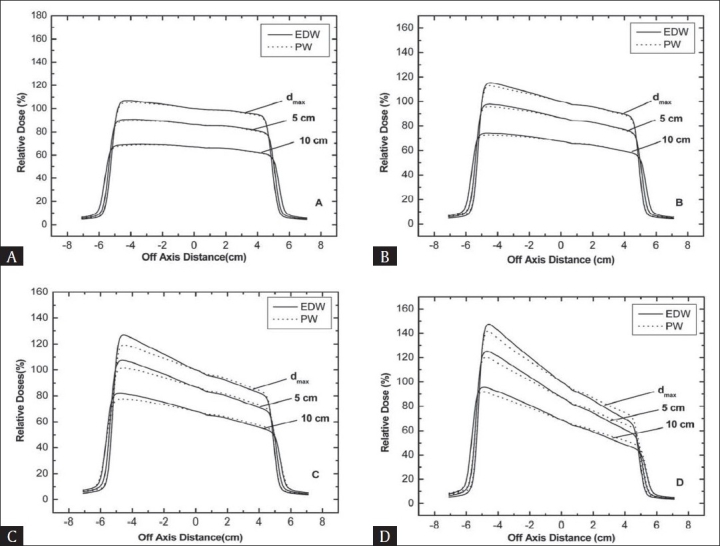
A comparison of wedge beam profiles at the depth of dmax, 5 and 10 cm for 6 MV beam with a field size of 10 ×10 cm^2^ (A) 15° wedges (B) 30° wedges (C) 45° wedges (D) 60° wedges.

**Figure 6:**
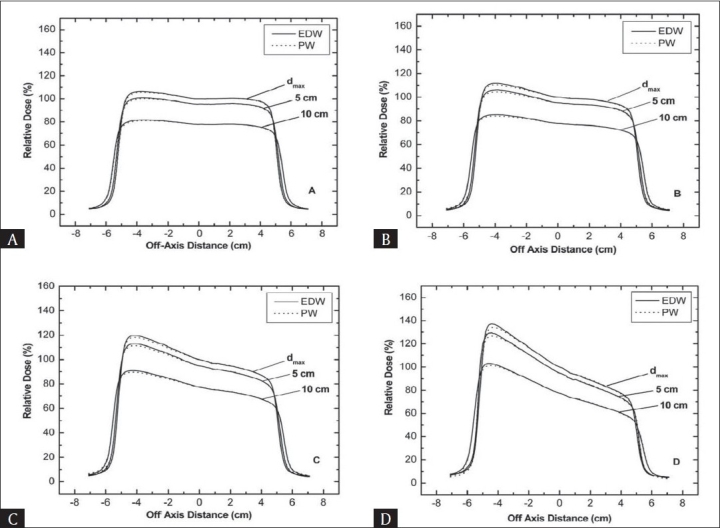
A comparison of wedge beam profiles at the depth of dmax, 5 and 10 cm for 15 MV beam with a field size of 10 ×10 cm^2^ (A) 15° wedges (B) 30° wedges (C) 45° wedges (D) 60° wedges.

Figures 5 and 6 show beam profiles in the wedge direction at d_max,_ 5 cm and 10 cm depths for both types of wedges in 6 and 15 MV beams respectively. The field size was kept constant at 10 × 10 cm^2^. The profiles were taken using LDA-99™ diode array having resolution less than 1 mm. The use of LDA-99 array is superior over other dose measuring devices specifically in sharp dose gradient regions (i.e. penumbra). The EDW dose profiles match will with the PW, except at the toe and heel region of thick wedges. At the toe side the relative doses for EDW are higher than that for PW, while at the heel side an inverse effect was observed. This relative difference between PW and EDW was higher at toe side compared to heel side. The difference decreases with increase in depth, while increases with increase in wedge angle especially for low energy photon beam.

With increasing depth, doses for both PW and EDW profiles were reduced due to reduced photon fluence. This is because in PW, low energy photons are preferentially absorbed in the wedge material and mean energy of beam after passing wedge is increased. Therefore variation in photon fluence with depth will be small for PW. However, for EDW there is no preferential absorption of low energy photons (no beam hardening effect). Therefore low energy photons will be present in the beam when it reaches the water surface. Hence the difference between PW and EDW profiles at shallower depths is high. This difference is decreased with increase in depth because at larger depths beam hardening effect in PW is compensated by the absorption of low energy photon in upper water layer in the EDW beam. With increasing depth the photon spectrum for both PW and EDW become almost equal for thin wedges and two profiles are quite similar.

**Figure 7:**
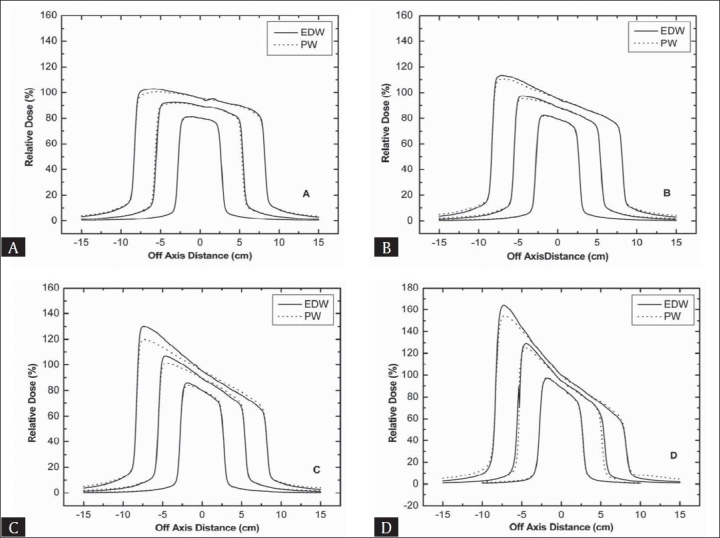
A comparison of wedge beam profiles at the field size of 5 × 5, 10 × 10 and 15 × 15 cm^2^ for 6 MV beam with a depth of 10 cm (A) 15° wedges (B) 30° wedges (C) 45° wedges (D) 60° wedges

**Figure 8:**
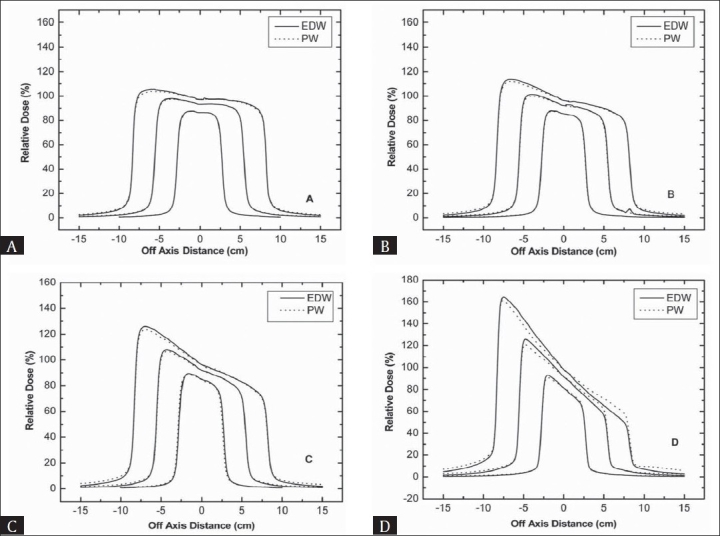
A comparison of wedge beam profiles at the field size of 5 × 5, 10 × 10 and 15 × 15 cm^2^ for 15 MV beam with a depth of 10 cm (A) 15° wedges (B) 30° wedges (C) 45° wedges (D) 60° wedges

Figures 7 and 8 represent the comparison of profiles for a range of square field sizes taken at 10 cm depth for 6 and 15 MV beam respectively. With increase in field size, almost similar effect was observed as with increase in depth. For the 45° and 60° wedge, it was found that EDW has higher peak in the toe area than PW especially for larger field size studied. Another important observation was a higher difference at the toe side for thicker wedges with 6 MV. For 15 MV beam, dose profiles matched very well with each other.

Increase in difference at toe side between two profiles with field size is due to the fact that portion of physical wedge in the beam path is increased with increase in field size and hence a stronger beam hardening effect was occurred for PW. Beam hardening is increased with increase in thickness from toe to heal side for PW in the beam path. Towards the toe side the doses for PW are smaller than EDW due to such beam hardening effect as with EDW no such effect is related.

At the heel side of wedged distribution, doses for EDW were slightly smaller compared to PW. Since in EDW collimator sweeping starts from open to closed position, a fraction of total monitor units is given before jaw starts moving. This fraction depends upon field size, wedge angle, monitor units and beam energy. Therefore, reduced doses in EDW at heel side mean that this side is irradiated for a fraction of time only.

## Conclusion

In this paper we have presented a comparison of WF and beam profiles for Varian's physical and enhanced dynamic wedges. The absolute values of WF are greatly differing from each other. PW shows a strong depth dependence of WF, while EDWF is independent of the depth of measurements due to the absence of beam hardening. The field size dependence of both type of wedges are showing an inverse behavior to each other. PW shows an increasing behavior while enhance dynamic wedge shows decreasing behavior with increasing field size. Our data also supports the results of Palta JR[9] and therefore a single WF measured at a reference field size may not be valid for all field size.

Although difficult in commissioning, EDW treatment modality offers a distinct advantage over conventional PW treatment modality because only field size dependency of WF is to be taken into account for treatment dose calculation while for PW the effect of field size as well as depth must be taken into account. The profiles for PW and EDW should also be measured to confirm the actual EDW angles for different field sizes and depth.
